# Perinatal determinants of growth trajectories in children born preterm

**DOI:** 10.1371/journal.pone.0245387

**Published:** 2021-01-28

**Authors:** Elizabeth A. Jasper, Hyunkeun Cho, Patrick J. Breheny, Wei Bao, John M. Dagle, Kelli K. Ryckman

**Affiliations:** 1 Department of Epidemiology, University of Iowa, Iowa City, IA, United States of America; 2 Department of Biostatistics, University of Iowa, Iowa City, IA, United States of America; 3 Department of Pediatrics, University of Iowa, Iowa City, IA, United States of America; Texas A&M University College Station, UNITED STATES

## Abstract

**Background:**

A growing amount of evidence indicates in utero and early life growth has profound, long-term consequences for an individual’s health throughout the life course; however, there is limited data in preterm infants, a vulnerable population at risk for growth abnormalities.

**Objective:**

To address the gap in knowledge concerning early growth and its determinants in preterm infants.

**Methods:**

A retrospective cohort study was performed using a population of preterm (< 37 weeks gestation) infants obtained from an electronic medical record database. Weight z-scores were acquired from discharge until roughly two years corrected age. Linear mixed effects modeling, with random slopes and intercepts, was employed to estimate growth trajectories.

**Results:**

Thirteen variables, including maternal race, hypertension during pregnancy, preeclampsia, first trimester body mass index, multiple status, gestational age, birth weight, birth length, head circumference, year of birth, length of birth hospitalization stay, total parenteral nutrition, and dextrose treatment, were significantly associated with growth rates of preterm infants in univariate analyses. A small percentage (1.32% - 2.07%) of the variation in the growth of preterm infants can be explained in a joint model of these perinatal factors. In extremely preterm infants, additional variation in growth trajectories can be explained by conditions whose risk differs by degree of prematurity. Specifically, infants with periventricular leukomalacia or retinopathy of prematurity experienced decelerated rates of growth compared to infants without such conditions.

**Conclusions:**

Factors found to influence growth over time in children born at term also affect growth of preterm infants. The strength of association and the magnitude of the effect varied by gestational age, revealing that significant heterogeneity in growth and its determinants exists within the preterm population.

## Introduction

The role of early-life factors and experiences in the causation of adult diseases has received additional attention since the creation of the Fetal Origins of Adult Disease (FOAD) hypothesis by Dr. David J.P. Barker in 1990 [1]. Initial research implicated low birth weight as a major risk factor for later adult disease, including obesity, metabolic syndrome, coronary events, and adult mortality [2–10]. Additional research has suggested that rapid postnatal weight gain is also independently associated with obesity and metabolic syndrome in childhood and adolescence [10]. Specifically, those born at the lowest birth weights, who experience rapid postnatal growth, are at the greatest risk for later life morbidity and mortality, suggesting that a mismatch between environments in the perinatal period and infancy or early childhood leads to molecular alterations that persist over the life course [11–13].

Preterm infants, born before 37 weeks completed gestation, often experience both low birth weight and rapid postnatal growth. These infants also have added, unique characteristics and challenges that place them at further increased risk of later life morbidity and mortality, including higher likelihood of being born via Cesarean section, prolonged hospitalizations, and possessing morbidities like interventricular hemorrhage (IVH), respiratory distress syndrome (RDS), and necrotizing enterocolitis (NEC) [14, 15]. The metabolic abnormalities described in children born preterm, such as abnormal glucose levels, hyperbilirubinemia, reduced insulin sensitivity, and increased blood pressure, are also linked to their early growth and can lead to failure to thrive in infancy [16]. In addition to their increased biological vulnerability, the challenges faced by infants born prematurely are often amplified by environmental factors. A major environmental factor is inadequate nutrition, which stems from the early, abrupt change from the constant nutrition provided by their mothers to intermittent nutrition. The effect of this nutritional transition is further exacerbated by their underdeveloped gastrointestinal tracks’ ineffective absorption of critical nutrients and issues with breastfeeding [17, 18]. With heightened biologic vulnerability, compounded by environmental factors, preterm infants are at increased risk for abnormal growth outcomes. As early life growth has been linked to obesity, neurocognitive and academic deficits, and other later life morbidities, understanding the growth of preterm infants is critical [14, 15, 19, 20].

With a growing body of evidence suggesting that growth during infancy and early childhood is also associated with later-life morbidity [21–24], research on factors affecting early life growth could aid in the identification of individuals at increased risk of abnormalities and development of interventions that would modify growth and prevent later life morbidity and mortality. However, research investigating growth trajectories in preterm infants and the factors that underlie them is lacking [25–30], with previous research either failing to include these infants, possessing insufficient power, and/or failing to separate this unique group from their term counterparts [31–34]. Additionally, though a heterogeneous group in terms of characteristics, exposures, and risk factors, preterm infants are often treated as a homogenous unit. With rates of preterm birth increasing in the United States and roughly one in ten infants now born preterm, exclusion of this population is problematic [35, 36]. This research sought to address these gaps in knowledge regarding early childhood growth and its determinants in preterm infants.

## Materials and methods

### Study population

This retrospective cohort study was performed to examine the relationship between perinatal factors and growth trajectories until roughly two years of age. The study population was obtained from the Iowa Perinatal Health Research Collaborative (IPHRC) database, a resource composed of longitudinal electronic health record (EHR) data on neonates born at or transferred into the University of Iowa Hospitals and Clinics (UIHC) within the first 28 days of life. EHR data within this database were provided by the Institute for Clinical and Translational Science (ICTS) Bioinformatics Core at UIHC.

Infants born on or prior to June 27, 2018 were considered for the study. Preterm infants alive at discharge were considered for inclusion. Infants with major congenital anomalies, defined according to the guidelines of the National Birth Defects Prevention Network, were excluded from the population (S1 Appendix), as these infants are likely to have distinct risk factors and growth trajectories compared to infants without such conditions. As atrial septal defects (ASDs) are primarily a developmental issue and often resolve on their own, infants with ASDs were included in the study population. Infants with ventricular septal defects (VSDs) were also included under the same reasoning. However, infants with severe VSDs, defined as those requiring surgical closure or cardiac catheterization and those discharged on Lasix, Captopril, and Digoxin, were excluded. In order to ensure information on delivery was available, infants who were transferred to UIHC after birth were excluded. Those born to mothers less than 18 years of age and infants with incomplete information on gestational age, birth weight, or biological sex were also excluded. The retrospective study was approved for a waiver of consent by the University of Iowa Institutional Review Board (IRB number: 201410743).

### Predictor variables

Factors with known associations with weight were investigated as potential explanatory variables. As there is no previous research on determinants of growth trajectories exclusively in preterm infants, additional variables were also utilized in an exploratory manner. These variables were selected based on theory and established connections to birth weight. Factors that are observed at higher frequencies in preterm infants and factors that are known to affect overall health of infants born preterm were also considered as potential determinants of early life growth.

Briefly, potential explanatory variables, including demographic and perinatal characteristics in addition to postnatal factors and clinical disorders, were obtained through EHRs for all children and their mothers. Previously validated algorithms based on International Classification of Diseases, ninth and tenth revision, diagnosis codes were used to identify maternal diabetes during pregnancy, chorioamnionitis, preeclampsia, hypertension during pregnancy, depression, bronchopulmonary dysplasia (BPD), IVH, NEC, patent ductus arteriosus (PDA), periventricular leukomalacia (PVL), RDS, retinopathy of prematurity (ROP), atrial septal defect, and ventricular septal defect [37, 38]. These algorithms were validated via medical chart abstraction in 813 randomly selected preterm infants and 194 randomly selected mothers. Other variables abstracted from the EHR included gestational age, birth weight, birth length, head circumference, singleton/multiple birth, maternal race and ethnicity, maternal insurance status at delivery, maternal age at birth, type of membrane rupture (artificial rupture (AROM), premature rupture (PROM), spontaneous rupture of membranes (SROM), unknown type of rupture of membrane), method of delivery (Cesarean section versus vaginal), hypoglycemia and/or hyperglycemia during the first day of life, hyperbilirubinemia, dextrose and insulin therapy as defined by National Drug Codes (NDC), maternal first trimester body mass index (BMI), intubation or positive pressure ventilation, total parenteral nutrition (TPN), and Apgar score at one minute of life. Hypoglycemia and hyperglycemia during the first day of life were defined using laboratory values. The most commonly used clinical definition was utilized, with blood glucose levels of less than or equal to 50 milligrams per deciliter (mg/dL) representing hypoglycemia and blood glucose levels greater than or equal to 155 mg/dL representing hyperglycemia. Year of birth was utilized to account for potential changes in clinical practice, while length of stay for initial birth hospitalization was used as an indicator for severity of illness and prematurity.

### Outcomes

Measures of children’s weight were obtained from the EHRs. Weight was transformed to weight z-scores (z-weight/weight-for-age), growth indicator measures of weight adjusted for a child’s age and sex, for analyses using measurements of children in the World Health Organization’s (WHO) Multicentre Growth Reference Study [39]. As all included infants were preterm, ages at measurement points were adjusted for gestational age prior to comparison to WHO References, as is standard clinical practice. Corrected postnatal age was obtained by subtracting an individual’s age at birth, in weeks, from 40 before subtracting this value from the child’s chronological postnatal age.

Though the Academy of Pediatrics recommends healthcare examinations during the first week of life, by one month, and at two, four, six, nine, 12, 15, 18, and 24 months of age [40], timing and number of weight measurements in this population varied. Measurements of weight obtained at and after discharge were utilized for outcomes in this study. Growth trajectories were modeled from discharge until the corrected age of two years (± three months). The main outcome of interest was z-weight trajectories.

### Statistical analysis

#### Growth trajectories

Linear mixed-effects modeling was used to estimate z-weight trajectories from discharge until two years corrected age. This methodology allows for intrasubject correlation of repeated measures on a subject and accounts for the unbalanced design in the number and timing of z-weight measurements for each subject [41, 42]. Restricted Maximum Likelihood was used to estimate model parameters with random intercepts and slope. As siblings and multiples made up a significant portion of the population (31.07%), a sensitivity analysis was performed with familial clustering in the entire cohort. Variables that were significant in the un-clustered analysis were included in the clustered model to test the hypothesis that clustering on family was unimportant.

As the risk of development of various conditions, such as ROP, PDA, and IVH, varies drastically by gestational age, additional linear mixed-effects models were created using gestational age categories. Infants were divided into the following groups based on gestational ages: late (34–36 weeks gestation), moderate (30-<34 weeks gestation), and extremely preterm (<30 weeks completed gestation). Gestational age groups were compared using analysis of variance (ANOVA) for continuous variables and Chi-Square or Fisher’s Exact tests for categorical variables. Growth trajectory models were also created for each subgrouping. Sensitivity analyses were also performed based on minimum length of follow-up, with mixed models created for those with at least six months, one, and two years of follow-up (S1 Appendix).

### Regression

Prior to the investigation of determinants of individual growth trajectories, multivariate imputation by chained equations (fully conditional specification) was performed for missing predictor data using the MICE package in R [43, 44]. Prior to imputation, roughly 25% of infants were missing information on birth length, while 34% were missing head circumference. Approximately 61% of infants had mothers whose first trimester BMI was not recorded (S1 Appendix).

After individual growth trajectories were obtained, linear regression was used to investigate the effects of perinatal factors on the growth of children. The outcome was defined as growth over time (slope in years). In the overall model, including all preterm children, predictors that could affect all infants, regardless of gestational age, were utilized. Predictors were first considered individually, before being used to create a multivariable model. Factors significantly (p-value <0.20) associated with growth trajectories in univariate analyses for all preterm infants were included in a model, in order to understand the joint effects of perinatal factors on growth. A p-value less than 0.20 was selected in order to capture factors that are known to be associated with growth and/or might be significantly associated with growth velocity in subpopulations of preterm infants. A sensitivity analysis was performed in the cohort, where variables with more than 5.00% imputed data were excluded from the joint model. The overall joint model, with imputed variables, was then applied to each gestational age subgroup. In extremely and moderately preterm infants who are at highest risk of certain neonatal morbidities, potential explanatory variables that vary based on gestational age at birth were added to the model to investigate the additional predictive value of these early-life conditions. All statistical analyses were conducted using SAS Version 9.4 (SAS Institute, Cary, NC) [45].

## Results

### Cohort characteristics

**Fig 1:**
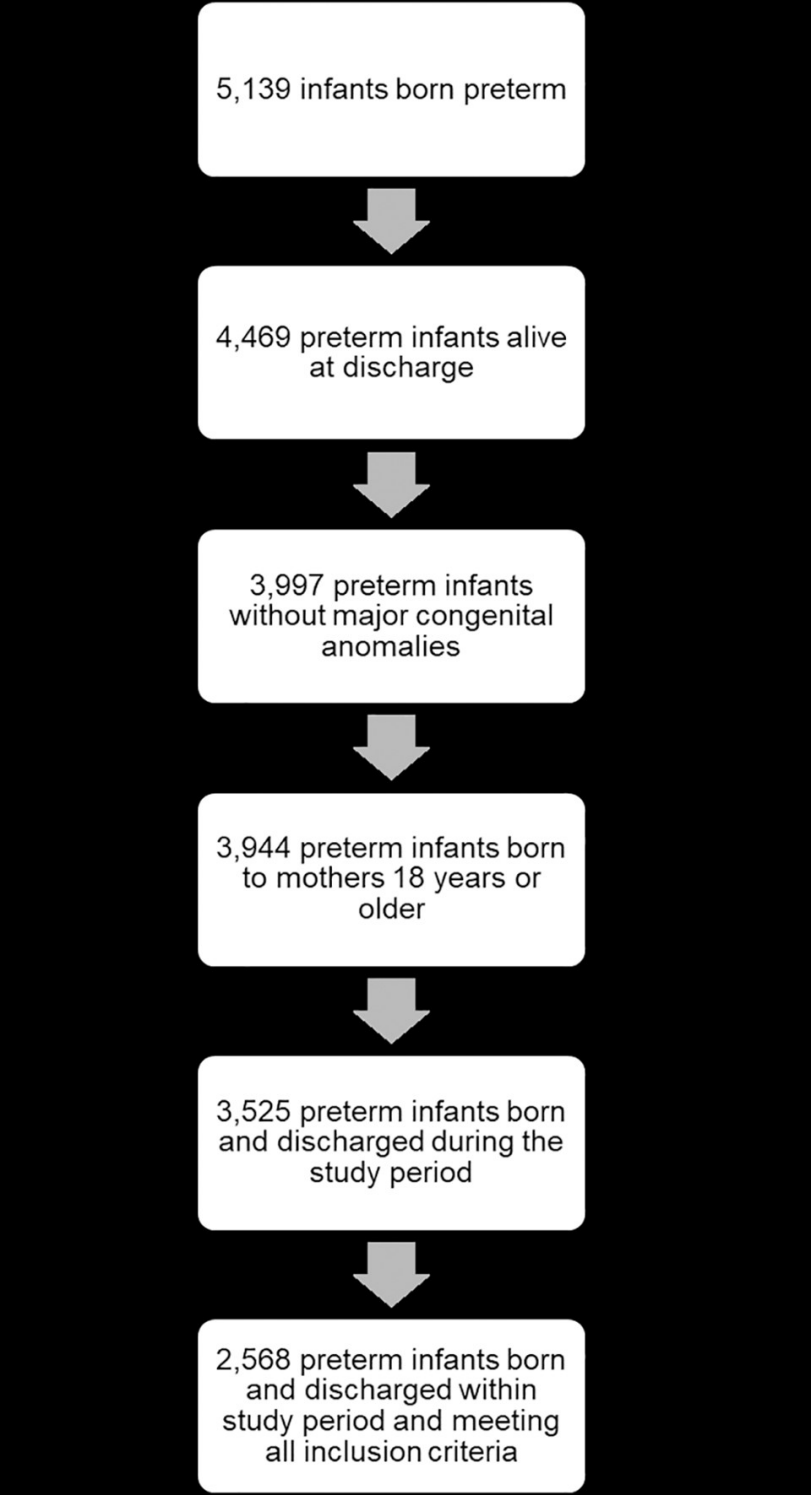
Study population.

Five thousand, one-hundred and thirty-nine infants were born preterm, with 4,469 surviving to discharge during the study period. There were 472 infants with major congenital anomalies who were excluded, while 53 were excluded for being born to mothers under the age of 18. Finally, 957 infants were excluded due to lack of follow-up after reaching a postnatal corrected age of 40 weeks. The study population included a total of 2,568 preterm newborns (Fig 1). Of these infants, 548 (21.34%) were classified as extremely preterm, 751 (29.24%) were moderately preterm, and the remaining 1,269 (49.42%) were late preterm. The demographic and clinical characteristics of the study population, after imputation, are shown in Table 1. Most infants were born to White, non-Hispanic or Latino mothers who had private/commercial or Medicaid insurance at delivery. Birth weight, length, head circumference, and Apgar score at one minute of life all increased as gestational age increased, with late preterm infants having, on average, larger birth weights, longer birth lengths, bigger head circumferences, and higher Apgar scores. In contrast, the length of stay for the birth hospitalization increased as the degree of prematurity increased, with extremely preterm infants having longer hospitalizations. The proportion of infants receiving TPN or suffering from major neonatal morbidities (BPD, IVH, etc.) also increased as the degree of prematurity increased.

**Table 1:** Characteristics of study population by degree of prematurity.

	Extremely Preterm (<30 weeks)	Moderately Preterm (30–33 weeks)	Late Preterm (34–36 weeks)	Total Population
	N = 548 (21.34%)	N = 751 (29.24%)	N = 1,269 (49.42%)	N = 2,568
	n (%)	n (%)	n (%)	n (%)
**Maternal characteristics and clinical factors**				
**Maternal race**				
**Asian**	16 (2.92%)	17 (2.26%)	54 (4.26%)	87 (3.39%)
**Black/African American**	75 (13.69%)	53 (7.06%)	109 (8.59%)	237 (9.23%)
**Multiple Race**	12 (2.19%)	10 (1.33%)	22 (1.73%)	44 (1.71%)
**Other**	35 (6.39%)	45 (5.99%)	99 (7.80%)	179 (6.97%)
**Unknown**	19 (3.47%)	16 (2.13%)	33 (2.60%)	68 (2.65%)
**White**	391 (71.35%)	610 (81.23%)	952 (75.02%)	1953 (76.05%)
**Maternal ethnicity**				
**Hispanic or Latino**	30–40 (<10%)^†^	50–60 (<10%)^†^	102 (8.04%)	187 (7.28%)
**Not Hispanic/Latino**	455 (83.03%)	647 (86.15%)	1103 (86.92%)	2205 (85.86%)
**Other**	<10 (<10%)^†^	<10 (<10%)^†^	19 (1.50%)	35 (1.36%)
**Unknown**	49 (8.94%)	47 (6.26%)	45 (3.55%)	141 (5.49%)
**Mother’s age (years)**^**‡**^	29.26 ± 5.96	29.76 ± 5.87	30.02 ± 5.66	29.78 ± 5.79
**Maternal primary insurance at delivery**				
**Medicaid**	187 (34.12%)	233 (31.03%)	339 (26.71%)	759 (29.56%)
**Medicare**	<10 (<15%)^†^	<10 (<10%)^†^	12 (0.95%)	22 (0. 68%)
**Other**	14 (2.55%)	23 (3.06%)	32 (2.52%)	69 (2.69%)
**Private**	285 (52.01%)	418 (55.66%)	755 (59.50%)	1458 (56.78%)
**Unknown**	60–70 (<15%)^†^	60–70 (<10%)^†^	131 (10.32%)	260 (10.12%)
**Maternal first trimester BMI**				
**Underweight**	<10 (<10%)^†^	<15 (<10%)^†^	23 (1.81%)	39 (1.52%)
**Normal BMI**	196 (35.77%)	296 (39.28%)	461 (36.33%)	952 (37.07%)
**Overweight**	<150 (<25%)^†^	<180 (<25%)^†^	327 (25.77%)	635 (24.73%
**Obese**	212 (38.69%)	272 (36.22%)	458 (36.09%)	942 (36.68%)
**Chorioamnionitis**	76 (13.87%)	44 (5.86%)	38 (2.99%)	158 (6.15%)
**Depression during pregnancy or prior to child’s discharge**	106 (19.34%)	131 (17.44%)	209 (16.47%)	446 (17.37%)
**Diabetes during pregnancy**	62 (11.31%)	143 (19.04%)	308 (24.27%)	513 (19.98%)
**Hypertension during pregnancy**	86 (15.69%)	216 (28.76%)	353 (27.82%)	655 (25.51%)
**Preeclampsia**	88 (16.06%)	233 (31.03%)	338 (26.64%)	659 (25.66%)
**Birth and neonatal characteristics**				
**Sex**				
**Female**	297 (54.20%)	339 (45.14%)	546 (43.03%)	1182 (46.03%)
**Male**	251 (45.80%)	412 (54.86%)	723 (56.97%)	1386 (53.97%)
**Birth year**				
**2009**	32 (5.84%)	44 (5.86%)	80 (6.30%)	156 (6.07%)
**2010**	60 (10.95%)	92 (12.25%)	132 (10.40%)	284 (11.06%)
**2011**	58 (10.58%)	57 (7.59%)	131 (10.32%)	246 (9.58%)
**2012**	58 (10.58%)	91 (12.12%)	157 (12.37%)	306 (11.92%)
**2013**	65 (11.86%)	81 (10.79%)	125 (9.85%)	271 (10.55%)
**2014**	63 (11.50%)	87 (11.58%)	160 (12.61%)	310 (12.07%)
**2015**	66 (12.04%)	93 (12.38%)	143 (11.27%)	302 (11.76%)
**2016**	72 (13.14%)	106 (14.11%)	137 (10.80%)	315 (12.27%)
**2017**	64 (11.68%)	82 (10.92%)	167 (13.16%)	313 (12.19%)
**2018**	10 (1.82%)	18 (2.40%)	37 (2.92%)	65 (2.53%)
**Multiples**	144 (26.28%)	278 (37.02%)	376 (29.63%)	798 (31.07%)
**Delivery Type**				
**Cesarean Section**	367 (66.97%)	486 (64.71%)	675 (53.19%)	1528 (59.50%)
**Vaginal**	181 (33.03%)	265 (35.29%)	594 (46.81%)	1040 (40.50%)
**Birth length (cm)**^**‡**^	34.51 ± 3.96	42.39 ± 3.47	46.83 ± 2.80	42.90 ± 5.78
**Birth weight (gr)**^**‡**^	951.61 ± 298.47	1747.17 ± 416.31	2536.43 ± 506.99	1967.42 ± 768.84
**Head circumference (cm)**^**‡**^	24.36 ± 2.75	29.69 ± 2.18	32.54 ± 1.73	29.96 ± 3.81
**1-minute Apgar score**^**‡**^	5 ± 2	6 ± 2	7 ± 2	6 ± 2
**Length of birth hospitalization stay (days)**^**‡**^	89.80 ± 40.18	36.36 ± 22.37	11.64 ± 10.55	35.55 ± 38.16
**Neonatal morbidities and treatments**				
**Atrial septal defects**	323 (58.94%)	136 (18.11%)	122 (9.61%)	581 (22.62%)
**BPD**	412 (75.18%)	75 (9.99%)	17 (1.34%)	504 (19.63%)
**Hypoglycemic**	243 (44.34%)	407 (54.19%)	690 (54.37%)	1340 (52.18%)
**Hyperglycemic**	215 (39.23%)	65 (8.66%)	32 (2.52%)	312 (12.15%)
**IVH**	97 (17.70%)	50–60 (<10%)^†^	<10 (<10%)^†^	154 (6.00%)
**NEC**	20 (3.65%)	10–20 (<10%)^†^	<10 (<10%)^†^	40 (1.56%)
**PDA**	280 (51.09%)	41 (5.46%)	29 (2.29%)	350 (13.63%)
**PVL**	22 (4.01%)	<10 (<10%)^†^	<10 (<10%)^†^	29 (1.13%)
**RDS**	514 (93.80%)	412 (54.86%)	363 (28.61%)	1289 (50.19%)
**ROP**	219 (39.96%)	10–20 (<10%)^†^	<10 (<10%)^†^	229 (8.92%)
**Ventricular septal defects**	22 (4.01%)	12 (1.60%)	26 (2.05%)	60 (2.34%)
**TPN**	536 (97.81%)	684 (91.08%)	256 (20.17%)	1476 (57.48%)

Abbreviations: BPD, bronchopulmonary dysplasia; IVH, intraventricular hemorrhage; NEC, necrotizing enterocolitis; PDA, patent ductus arteriosus; PVL, periventricular leukomalacia; RDS, respiratory distress syndrome; ROP, retinopathy of prematurity; TPN, total parenteral nutrition; gr, grams; cm, centimeters.

^†^Values given as a range, as data is suppressed due to counts less than 10 or to protect confidentiality.

^**‡**^Data are expressed as mean ± standard deviation.

Approximately 26,338 weight measurements were included in creation of z-weight trajectories for the entire study population. The minimum number of weight measurements a child had was one (308 individuals); while the maximum number of measurements was 202. The average number of weight measurements per child was 10. Extremely preterm infants had, on average, a larger number of measurements (N = 13) compared to moderately (N = 7) and late (N = 10) preterm infants. The average length of follow-up in the study population was 485.57 days (1.33 years). Like the number of measurements, extremely preterm infants had, on average, longer lengths of follow-up (1.48 years) than moderately (1.23 years) and late (1.32 years) preterm infants.

### Predictors of growth trajectories

In univariate analyses for all preterm infants, 13 variables were significantly (p-value < 0.20) associated with growth trajectories. These variables included sociodemographic factors (maternal race), maternal clinical conditions occurring during pregnancy (hypertension, preeclampsia, first trimester BMI group), neonatal characteristics (multiple gestation, gestational age, birth weight, birth length, head circumference, year of birth, length of birth hospitalization), and interventions/treatments (TPN, dextrose treatment). In a joint effects model, these factors explained roughly 2.02% of the variation in growth trajectories until two years corrected age (Table 2). Maternal race (p-value = 0.0023), gestational age (p-value = 0.0049), and dextrose treatment (p-value = 0.0316), remained significant in the joint effects model. Being born to an Asian mother was associated with a deceleration in growth post-discharge until two years corrected age. Specifically, preterm infants born to Asian mothers had a 0.16 decelerated rate of growth in z-weight per year compared to infants born to White mothers. In contrast, receiving dextrose during initial birth hospitalization and increasing gestational ages were associated with accelerated growth. Infants who received dextrose nutritional support had a 0.04 z-score acceleration in their growth rate per year compared to infants who did not receive such nutrition interventions. Finally, for each week increase in gestational age, the growth rate accelerated by 0.02 per year until two years corrected age. The change in weight z-score per year for variables in the joint model for all preterm infants can be viewed in Table 2.

**Table 2:** Multivariable model with and without clustering in all preterm infants.

	Un-clustered			Clustered on familial relation		
Variable	Parameter estimate(s)	SE	P-value	Parameter estimate(s)	SE	P-value
**Intercept**	11.02142	5.68115	0.0525	10.60334	5.63184	0.0598
**Maternal race**						
**Asian**	-0.15719	0.03980	<0.0001*	-0.15903	0.03946	<0.0001*
**Black/African American**	0.00283	0.02518	0.9105	0.00383	0.02496	0.8779
**Multiple races**	-0.09668	0.05513	0.0796	-0.09575	0.05465	0.0799
**Other**	-0.00151	0.02850	0.9579	-0.00227	0.02826	0.9361
**Unknown**	-0.02251	0.04450	0.6130	-0.01815	0.04412	0.6808
**Maternal first trimester BMI**						
**Underweight**	-0.10882	0.05894	0.0650	-0.11562	0.05843	0.0479
**Overweight**	0.01170	0.01860	0.5294	0.00848	0.01843	0.6456
**Obese**	0.03154	0.01762	0.0736	0.02956	0.01747	0.0908
**Maternal hypertension during pregnancy**	-0.00294	0.01909	0.8777	-0.00360	0.01892	0.8492
**Preeclampsia**	0.00653	0.01859	0.7256	0.00851	0.01843	0.6444
**Birth year**	-0.00552	0.00282	0.0507	-0.00531	0.00280	0.0578
**Multiple(s)**	0.02730	0.01593	0.0867	0.03036	0.01579	0.0547
**Gestational age (weeks)**	0.01599	0.00569	0.0049*	0.01543	0.00564	0.0062*
**Birth weight (gr)**	-0.00002	0.00003	0.4994	-0.00002	0.00003	0.5108
**Birth length (cm)**	-0.00022	0.00337	0.9469	0.00008	0.00334	0.9802
**Head circumference (cm)**	-0.00915	0.00529	0.0837	-0.00894	0.00524	0.0884
**TPN**	-0.02370	0.02119	0.2634	-0.02204	0.02100	0.2941
**Received dextrose**	0.03910	0.01818	0.0316*	0.03780	0.01802	0.0361*
**Length of birth hospitalization stay (days)**	-0.00029	0.00037	0.4378	-0.00029	0.00037	0.4307

Abbreviations: BMI, body mass index; cm, centimeters; gr, grams; SE, standard error; TPN, total parenteral nutrition.

Referent groups: White maternal race, normal maternal first trimester BMI, no hypertension, no preeclampsia, singleton, no TPN, no dextrose.

*p<0.05. P-values were calculated using multivariable linear regression.

Clustering on family did not have a significant effect on the model, as the variables found to be significant in the joint model were virtually the same as in the un-clustered model. Most parameter estimates changed by less than 20.00% when clustering on familial relation (Table 2). Furthermore, significant associations remained after clustering on family. As such, all other models were obtained without clustering. When variables that were imputed for more than 5.00% of the population (maternal first trimester BMI, birth length, and head circumference) were excluded from the joint model, the percent of variation in growth trajectories that was explained by the model decreased by 0.30% (Table 3). Though this suggests a small portion of the variation in growth over time is explained by these factors, the true importance of these variables cannot be evaluated separately due to their imputation. Nevertheless, factors found to significantly influence growth over time in the original model, including maternal race, gestational age, and receiving dextrose, remained significant in the model that excluded the imputed variables.

**Table 3:** Multivariable model compared to model excluding significantly imputed variables.

	Final model			Final model excluding imputed variables		
Variable	Parameter estimate(s)	SE	P-value	Parameter estimate(s)	SE	P-value
**Intercept**	11.02142	5.68115	0.0525	10.68852	5.67347	0.0597
**Maternal race**						
**Asian**	-0.15719	0.03980	<0.0001*	-0.16569	0.03971	<0.0001*
**Black/African American**	0.00283	0.02518	0.9105	0.01100	0.02500	0.6599
**Multiple races**	-0.09668	0.05513	0.0796	-0.08903	0.05504	0.1059
**Other**	-0.00151	0.02850	0.9579	0.00601	0.02837	0.8322
**Unknown**	-0.02251	0.04450	0.6130	-0.01811	0.04452	0.6841
**Maternal first trimester BMI**						
**Underweight**	-0.10882	0.05894	0.0650	NA	NA	NA
**Overweight**	0.01170	0.01860	0.5294			
**Obese**	0.03154	0.01762	0.0736			
**Maternal hypertension during pregnancy**	-0.00294	0.01909	0.8777	0.00664	0.01841	0.7182
**Preeclampsia**	0.00653	0.01859	0.7256	0.00738	0.01860	0.6917
**Birth year**	-0.00552	0.00282	0.0507	-0.00538	0.00282	0.0564
**Multiple(s)**	0.02730	0.01593	0.0867	0.02301	0.01579	0.1452
**Gestational age (weeks)**	0.01599	0.00569	0.0049*	0.01040	0.00506	0.0401*
**Birth weight (gr)**	-0.00002	0.00003	0.4994	-0.00004	0.00002	0.0553
**Birth length (cm)**	-0.00022	0.00337	0.9469	NA	NA	NA
**Head circumference (cm)**	-0.00915	0.00529	0.0837	NA	NA	NA
**TPN**	-0.02370	0.02119	0.2634	-0.03083	0.02076	0.1376
**Received dextrose**	0.03910	0.01818	0.0316*	0.03964	0.01811	0.0287*
**Length of birth hospitalization stay (days)**	-0.00029	0.00037	0.4378	-0.00023	0.00037	0.5365

Abbreviations: BMI, body mass index; cm, centimeters; gr, grams; SE, standard error; TPN, total parenteral nutrition.

Referent groups: White maternal race, normal maternal first trimester BMI, no hypertension, no preeclampsia, singleton, no TPN, no dextrose.

*p<0.05. P-values were calculated using multivariable linear regression.

### Stratified analyses

In analyses stratified by gestational age, the significance and strength of associations between variables and growth trajectories differed based on gestational age (Table 4). The percent of variation in z-weight growth explained by the model also varied based on degree of prematurity, with 1.60%, 2.70%, and 1.32% of change in z-weight explained by these 13 perinatal factors in extremely, moderately, and late preterm infants, respectively. There was not a perinatal factor was significantly associated with (z-weight) growth rate in every stratum. In extremely preterm infants, maternal race and receiving dextrose nutritional support remained significant in the joint model. However, the significance of maternal race in this population was driven by those born to multi-race mothers, with these infants having a 0.32 deceleration in z-weight change per year compared to infants born to White mothers. Dextrose treatment was also significant in extremely preterm infants, though the magnitude of the effect was larger in this group, resulting in infants given dextrose having a 0.11 acceleration in z-weight growth per year in the first two years of life.

**Table 4:** Multivariable model applied to gestational age strata.

	Extremely preterm			Moderately preterm			Late preterm		
Variable	Parameter estimate	SE	P-value	Parameter estimate	SE	P-value	Parameter estimate	SE	P-value
**Intercept**	15.41590	18.38236	0.4021	5.30311	5.14653	0.3032	13.74418	8.64866	0.1123
**Maternal race**									
**Asian**	-0.21607	0.13406	0.1076	-0.06529	0.04333	0.1322	-0.16059	0.05337	0.0027*
**Black/African American**	0.02864	0.06822	0.6748	0.00834	0.02529	0.7417	-0.02320	0.03864	0.5483
**Multiple races**	-0.31986	0.15429	0.0386*	-0.05238	0.05542	0.3449	-0.04972	0.08167	0.5428
**Other**	-0.03428	0.09389	0.7152	0.00854	0.02739	0.7553	0.00458	0.04051	0.9100
**Unknown**	0.04594	0.12294	0.7088	-0.06132	0.04392	0.1631	0.01390	0.06709	0.8359
**Maternal first trimester BMI**									
**Underweight**	-0.23952	0.21672	0.2696	-0.07126	0.05621	0.2053	-0.08862	0.08089	0.2735
**Overweight**	-0.00045	0.05970	0.9939	0.01503	0.01677	0.3704	0.01341	0.02756	0.6268
**Obese**	-0.04356	0.05627	0.4392	0.01632	0.01533	0.2874	0.05917	0.02665	0.0266*
**Maternal hypertension during pregnancy**	0.07402	0.07363	0.3153	-0.02503	0.01558	0.1085	0.00472	0.02840	0.8680
**Preeclampsia**	0.01380	0.07472	0.8535	0.02534	0.01554	0.1034	-0.01438	0.02771	0.6039
**Birth year**	-0.00732	0.00913	0.4232	-0.00263	0.00256	0.3047	-0.00752	0.00430	0.0803
**Multiple(s)**	0.01257	0.05224	0.8099	0.04351	0.01375	0.0016*	0.01448	0.02452	0.5549
**Gestational age (weeks)**	0.00611	0.02269	0.7876	0.01201	0.00765	0.1170	0.03424	0.01678	0.0415*
**Birth weight (gr)**	0.00022	0.00017	0.2021	-0.00002	0.00003	0.5541	-0.00007	0.00004	0.0719
**Birth length (cm)**	-0.01012	0.01085	0.3512	-0.00083	0.00275	0.7633	0.01086	0.00572	0.0579
**Head circumference (cm)**	-0.02426	0.01849	0.1902	-0.00702	0.00404	0.0828	-0.00104	0.00912	0.9092
**TPN**	-0.10297	0.17977	0.5670	0.00248	0.02417	0.9183	-0.01394	0.03343	0.6766
**Received dextrose**	0.11358	0.05122	0.0270*	0.01285	0.02366	0.5873	0.03330	0.02801	0.2347
**Length of birth hospitalization stay (days)**	-0.00040	0.00086	0.6425	-0.00031	0.00032	0.3254	0.00155	0.00150	0.3030

Abbreviations: BMI, body mass index; cm, centimeters; gr, grams; SE, standard error; TPN, total parenteral nutrition.

Referent groups: White maternal race, normal maternal first trimester BMI, no hypertension, no preeclampsia, singleton, no TPN, no dextrose.

*p<0.05. P-values were calculated using multivariable linear regression.

In moderately preterm infants, the major significant perinatal factor influencing growth rates was multiple gestation (p-value = 0.0016). Twins and triplets had a 0.04 acceleration in z-weight growth per year during their first two years of life compared to singletons. Finally, in late preterm infants, maternal race, maternal first trimester BMI, and gestational age had the most significant effects on a child’s growth over time. Being born to an Asian mother was again associated with deceleration in growth. The magnitude of deceleration was almost the same as the magnitude of effect in the entire population. Unlike other strata, maternal first trimester BMI was found to impact children’s rate of growth until two years corrected age. The significance of this perinatal factor was largely due to the obese group. Infants of obese mothers had a 0.06 increase in the rate of z-weight change compared to infants born to mothers with normal first trimester BMIs. In the joint, fully adjusted models, maternal hypertension, preeclampsia, year of birth, birth weight, birth length, head circumference, TPN, and length of stay lost their significance in all strata after accounting for all other variables (Table 4). While differences among strata were detected, caution should be taken as the power to detect these effects vary by strata.

### Extremely and moderately preterm infants: major morbidities

In extremely and moderately preterm infants, the addition of conditions whose risk differs by the degree of prematurity increased the percent of variation in growth that was explained by the model (Table 5). An additional 2.08% of the variation in growth rates was explained in extremely preterm infants when the 9 conditions were included in the model (adjusted R^2^ = 3.68%). Of these conditions, PVL and ROP had the most profound, long-term impact on growth. Infants who suffered from PVL (-0.28 z-score per year) and ROP (-0.12 z-score per year) had worse rates of growth compared to those without such conditions. In moderately preterm infants, who have a decreased risk of these ailments, no condition was significantly associated with changes in rate of growth through infancy and early childhood. Furthermore, the percent of variation in the rate of growth explained by the model did not significantly increase when these conditions were incorporated in the moderately preterm strata (Table 5).

**Table 5:** Extremely and moderately preterm models with prematurity-associated conditions.

	Extremely preterm			Moderately preterm		
Variable	Parameter estimate(s)	SE	P-value	Parameter estimate(s)	SE	P-value
**Intercept**	19.65406	18.58351	0.2907	5.16553	5.46977	0.3453
**Maternal race**						
**Asian**	-0.21821	0.13315	0.1018	-0.06379	0.04363	0.1442
**Black/African American**	0.00894	0.06816	0.8957	0.01126	0.02542	0.6578
**Multiple races**	-0.39875	0.15481	0.0103*	-0.05709	0.05676	0.3149
**Other**	0.04129	0.09334	0.6584	0.01140	0.02757	0.6794
**Unknown**	0.03405	0.12208	0.7804	-0.06567	0.04467	0.1420
**Maternal first trimester BMI**						
**Underweight**	-0.25808	0.21640	0.2336	-0.06826	0.05664	0.2286
**Overweight**	0.00252	0.05995	0.9665	0.01282	0.01687	0.4474
**Obese**	-0.03903	0.05690	0.4931	0.01522	0.01546	0.3253
**Maternal hypertension during pregnancy**	0.06032	0.07347	0.4120	-0.02297	0.01573	0.1446
**Preeclampsia**	-0.00084	0.07457	0.9910	0.02373	0.01568	0.1307
**Birth year**	-0.00928	0.00923	0.3152	-0.00256	0.00272	0.3478
**Multiple(s)**	-0.00620	0.05308	0.9070	0.04542	0.01385	0.0011*
**Gestational age (weeks)**	-0.00583	0.02319	0.8017	0.01111	0.00784	0.1572
**Birth weight (gr)**	0.00022	0.00017	0.1896	-0.00001	0.00003	0.6618
**Birth length (cm)**	-0.01210	0.01081	0.2635	-0.00083	0.00277	0.7645
**Head circumference (cm)**	-0.02160	0.01841	0.2414	-0.00696	0.00407	0.0879
**TPN**	-0.03667	0.18426	0.8423	0.00185	0.02443	0.9397
**Received dextrose**	0.11587	0.05107	0.0237*	0.01456	0.02384	0.5416
**Length of birth hospitalization stay (days)**	-0.00049	0.00088	0.5749	-0.00031	0.00034	0.3673
**BPD**	0.04352	0.06179	0.4815	-0.01114	0.02341	0.6343
**IVH**	-0.05261	0.06085	0.3876	0.00217	0.02553	0.9323
**NEC**	0.08422	0.12069	0.4856	0.03182	0.04968	0.5220
**PDA**	-0.00120	0.05181	0.9815	0.00969	0.03110	0.7554
**PVL**	-0.28464	0.11648	0.0149*	-0.00768	0.07944	0.9231
**RDS**	-0.00964	0.09823	0.9218	-0.00429	0.01409	0.7609
**ROP**	-0.11541	0.05215	0.0273*	0.10869	0.05649	0.0545
**Atrial septal defect**	0.02489	0.04802	0.6045	-0.00311	0.01872	0.8681
**Ventricular septal defect**	0.22274	0.11576	0.0549	0.06055	0.05134	0.2386

Abbreviations: BMI, body mass index; BPD, bronchopulmonary dysplasia; cm, centimeters; gr, grams; IVH, intraventricular hemorrhage; NEC, necrotizing enterocolitis; PDA, patent ductus arteriosus; PVL, periventricular leukomalacia; RDS, respiratory distress syndrome; ROP, retinopathy of prematurity; TPN, total parenteral nutrition

Referent groups: White maternal race, normal maternal first trimester BMI, no hypertension, no preeclampsia, singleton, no TPN, no dextrose, no BPD, no IVH, no NEC, no PDA, no PVL, no RDS, no ROP, no atrial septal defect, no ventricular septal defect.

*p<0.05. P-values were calculated using multivariable linear regression.

## Discussion

In the past few decades, survival of preterm infants has radically improved with advances in medical care [46]. However, these infants are at increased risk of significant morbidities, including postnatal growth failure. As postnatal growth failure is believed to impact cognitive and motor development in childhood and place these infants at heightened risk of disease in adolescence and adulthood [19, 47, 48], understanding factors that influence growth in infants who are highly susceptible to growth failure, including preterm infants, is crucial. In this retrospective longitudinal study, early growth of this understudied population was evaluated. Factors found to influence growth over time in term infants, such as maternal race, gestational age, and multiple gestations, were found to influence growth in preterm infants too. Factors that are more common in preterm infants, such as receiving dextrose support, ROP, and PVL, also affected growth rates.

Gestational age was a significant determinant of children’s growth rates in the overall model, suggesting that growth rates differ based on the degree of prematurity. This observation lends further support to the notion that preterm infants are a heterogenous group in terms of both their physiological and structural immaturity, as well as their exposures and varying risks of morbidity and mortality [49–51]. The differences in characteristics and exposures observed between the preterm categories provide additional confirmation that a large degree of heterogeneity exists within the preterm population. In the model using all infants, every week increase in gestational age resulted in acceleration of growth after discharge. There are numerous possible mechanisms that could explain this finding. Infants who have spent longer time in utero have more time to develop in a protected environment with continuous nutrient intake. Infants born earlier may not receive the adequate, continuous nutrition that they would have in utero, potentially resulting in a slower rate of growth in early life. Compared to term infants, preterm infants also have a slower progressive increase in their metabolic rate after delivery [52, 53]. Late preterm infants are born less developmentally immature and have lower risks of major neonatal morbidities and complications [54]. Furthermore, older preterm infants often spend less time in neonatal intensive care units (NICUs), a location where they could have additional exposures that would have adverse effects on growth. Interestingly, in stratified analyses, gestational age was most significant in the late preterm group. Unsurprisingly, infants born closer to term have the largest acceleration in growth rates. The biologic mechanism responsible for accelerated growth rates in these older preterm infants could again be due to differences in developmental maturity and exposures in the perinatal period.

Previous research has estimated roughly half of the variation in postnatal growth is related to nutritional (energy and protein) intake [55]. Our study demonstrated a significant association between early nutritional support, in the form of dextrose, and growth rates in a preterm population. Infants that received dextrose during their NICU stay had accelerated growth after discharge compared to those who did not receive this form of nutrition. Increased growth rates in infants given this nutritional support has a clear molecular mechanism. Preterm infants often have limited glycogen stores but high energy requirements, which are often not met through standard term infant feeding practices. Furthermore, the total caloric intake devoted to facilitating growth is highest in the perinatal period. If energy intake dips below basal metabolic requirements, growth and the energy expenditure for these processes will be reduced. As the main source of (dietary) energy, receiving additional glucose (dextrose) enables these infants to spend more energy on growth and, likely, results in accelerated growth. Receiving dextrose was most significant in the extremely preterm population, a group particularly at risk of early nutrient deficiencies. Again, the provision of additional glucose likely prevents or lessens early energy deficiencies and allows infants to spend an appropriate or added amount of energy on growth. Dextrose, however, does not provide infants with the complete nutrition necessary for growth. TPN, in contrast, provides complete nutrition and includes dextrose. The finding of dextrose being a significant predictor of growth trajectories, while TPN lacked significance, was unexpected. However, this could be attributable to clinical practices and comparison group sizes. The vast majority of the extremely preterm infants received TPN. Lack of statistical significance is likely because most infants in this group received the intervention, prohibiting our ability to accurately gauge the impact of TPN in this group. The opposite is true of the late preterm group: late preterm infants rarely received TPN, thus there was not an appropriately sized group of late preterm infants who received TPN to compare these infants to. Further research on the contributions of neonatal nutrition, including forms not readily available in the electronic health records (e.g. breast milk), is necessary to gauge their impact on growth in preterm populations.

Maternal race was a significant determinant of preterm infants’ growth rates. The significance of maternal race was largely powered by the Asian group, with infants born to these mothers experiencing a deceleration in growth compared to infants born to White women. This deceleration in growth mirrors results seen in previous studies of growth in children born at term, which have found racial differences in fetal growth, early life BMI trajectories, and size obtained later in life [56–58]. Though the effects of being born to Black mothers did not reach statistical significance, the direction of the relationships also aligns with previous literature [57, 58]. Maternal race was also a significant determinant of early life growth in gestational age strata. In late preterm infants, a deceleration in infants born to Asian mothers compared to White mothers was also seen. However, in extremely preterm infants, those born to mothers who identified as multi-race had the most significant deviation from the referent group’s (White) growth rate. These infants experienced a drastic deceleration in (z-weight) growth. Although differences in the growth of racial and ethnic groups were seen in this study and have been observed in other research, the Centers for Disease Control and Prevention (CDC) currently promotes using a single set of growth charts for all individuals, regardless of their race or ethnicity. This recommendation is based on research which suggests that the basis for these differences in growth are largely the result of environmental factors, like the physical and geographical surroundings, social environment, relationships with family and peers, and nutrition, rather than genetic influences [59–61]. Further research is also necessary to determine the exact mechanisms responsible for differences in growth rates between racial and ethnic groups, including examination of the effects of systematic racism in the healthcare system and equal access to care. The influence of maternal race in this study should also be viewed with caution, as the effects reaching statistical significance were in groups that made up a very small proportion of the study population. Future studies with more diverse populations would enable verification or contradiction of these results.

A key finding of the study focused on extremely preterm infants. In these individuals, conditions associated with prematurity, though often thought of as short-term problems, had lasting long-term consequences on growth. PVL was the condition most strongly related to a child’s growth, with infants suffering from PVL having a 0.28 deceleration in growth rate per year compared to infants without this cranial abnormality. By age two, these infants would be, on average, over a half a standard deviation z-score smaller than their unaffected peers. PVL has previously been associated with smaller head circumferences and lower weight and length [62, 63]. It was also found to be a significant predictive factor for higher risk of growth failure in preterm infants around two years of age [64]. Our findings further support PVL’s link to poor growth outcomes. The mechanism underscoring this cranial abnormality and poor postnatal growth outcomes is unknown. However, infants with PVL also experience impaired brain growth as they age [65]. With the brain playing a vital role in the regulation of metabolism and growth, the simplest explanation for this finding is that injury and impaired brain growth result in the brain’s diminished ability to control pathways related to physical growth and development. As several risk factors, including maternal obesity and chorioamnionitis, for PVL are also known risk factors for poor growth, another possible explanation for these findings is that PVL may simply represent another consequence of perinatal factors or a mediator on the causal pathway between these factors and poor growth [66]. Finally, oral feeding difficulties, and, therefore, inadequate nutritional intake has been observed in animal models and infants with this conditions [67, 68]. Additional research is necessary in order to verify the relationship between PVL and poor growth rates in extremely preterm infants and to establish the biologic mechanisms responsible for this association.

Retinopathy of prematurity was also a significant determinant of growth rates in extremely preterm infants. Infants with ROP, like those with PVL, also experienced a deceleration in growth rate compared to those without the eye condition. Specifically, infants with ROP experienced, on average, a 0.12 deceleration in the rate of (z-score) growth per year compared to those without ROP, resulting in infants with ROP being, on average, roughly a fourth of a standard deviation z-score smaller than those without ROP by two years corrected age. The biologic mechanism linking poor growth rates to ROP is unclear. Poor postnatal weight gain in the first few weeks of life, when most preterm infants are still hospitalized, has previously been identified as a risk factor for development of ROP [69, 70]. Poor nutrition, such as lower receipt of lipids, calories, and carbohydrates, has also been observed at greater frequency in infants with ROP [71]. Thus, ROP may share important risk factors (poor growth in the hospital and poor nutrition) with poor rates of growth in early life. Additionally, development of ROP is strongly associated with low serum insulin-like growth factor 1 (IGF-1), a somatic growth factor essential for the normal growth and development of multiple tissues, such as the brain and blood vessels [72]. The IGF-1 hormone mediates the growth promoting influence of growth hormone (GH), in addition to exerting its own independent growth stimulating effects [73]. ROP may be an additional consequence or an indicator of early alterations in these molecular pathways and metabolism. However, additional research is necessary to verify the role of ROP in the causal pathway leading to poor later life growth outcomes and to determine the true biologic mechanism linking ROP to poor rates of growth in extremely preterm infants.

There are several limitations to this study. As a secondary data analysis, the inclusion of every important abnormal growth risk and protective factor was not possible. Dietary variables after discharge, including breastfeeding and timing of introduction of solid food, social variables (parental education, family income, etc.), and physical activity was not reliably found in medical records or accounted for in analyses. However, the primary goal of this study was to identify determinants of growth trajectories based on variables available immediately at birth and already collected during routine clinical care. This approach could enable the identification of individuals at increased risk of abnormal growth outcomes. While a moderate amount of missing data was present, multiple imputation was performed and a sensitivity analysis excluding variables that were imputed demonstrated little change in the level of variation in growth trajectories that was explained or in the significance of the predictors. The study population consisted primarily of infants born to White, Non-Hispanic mothers who had insurance. Therefore, generalizability of these results is limited. Future studies on early life determinants of growth trajectories in preterm infants should aim to include a more diverse population that better reflects the larger U.S. population.

The study has several strengths. First, repeated measurements of body size during infancy and early childhood were utilized, while the correlation between such data points and the unbalanced nature of the data was accounted for by using mixed-effects modeling. By using EHR data, there was information on a wide range of relevant characteristics and clinical factors for analysis. The study was also strengthened by using validated algorithms to define potential explanatory variables. Furthermore, the accuracy of our electronic database and algorithms for coding maternal and neonatal conditions were verified on a portion of the data by medical record abstraction. The outcome (growth trajectories) was created by using standardized growth indicators, z-weights, which accounts for the difference in growth by gender and age. These indicators have the advantage of allowing visualization of individual growth while also referencing a population standard [46]. Z-scores also better accommodate extreme weight values, which can often be observed in preterm infants. By using the WHO growth standards, preterm infants were compared to healthy, term infants who grew in the optimal environments and conditions. Furthermore, all weight measures were obtained from EHR data. This method of assessment is likely more reliable than self-reported weights.

This study found that established determinants of growth in children born at term also affect growth of preterm infants. Factors that occur at higher frequency in preterm infants also exert significant influence on growth rates. Interestingly, common complications of prematurity, often believed to be short-term issues, had long-term effects on growth of extremely premature infants. In summary, factors at birth and prior to discharge from the hospital influence a portion of growth in the early life of preterm infants, further supporting the Barker hypothesis and displaying a need for added research and interventions targeting the perinatal period. Future research should further characterize the growth of preterm infants. Growth trajectories can then be linked to later life outcomes to better understand the associations between the perinatal period and later life morbidities in individuals born prematurely.

## Supporting information

S1 Appendix (DOCX)
