# Supplementary material for: Perinatal determinants of growth trajectories in children born preterm
Source: PLoS One. 2021 Jan 28;16(1):e0245387. doi: 10.1371/journal.pone.0245387 (PMC7842887; doi:10.1371/journal.pone.0245387)
Supplement: S1 Appendix — (DOCX) [file pone.0245387.s001.docx]

Table 1. National Birth Defects Registry Definition of Major Birth Defects

| **Birth Defect** | **ICD-9-CM Codes** | **ICD-10-CM Codes** |
| --- | --- | --- |
| **CNS** |  |  |
| **Anencephalus** | 740.0x — 740.1x | Q00.0x — Q00.1x |
| **Spina bifida without**  **anencephalus** | 741.0x, 741.9x w/o 740.0x, 740.10x | Q05.0x — Q05.9x, Q07.01, Q07.03 |
| **Encephalocele** | 742.0x | Q01.0x — Q01.9x |
| **Holoprosencephaly** | 742.2x | Q04.2 |
| **Eye** |  |  |
| **Anophthalmia/microphthalmia** | 743.0x, 743.1x | Q11.0x — Q11.2x |
| **Congenital cataract** | 743.30x — 743.34x | Q12.0x |
| **Ear** |  |  |
| **Anotia/microtia** | 744.01x, 744.23x | Q16.0x, Q17.2x |
| **Cardiovascular** |  |  |
| **Aortic valve stenosis** | 746.3x | Q23.0x |
| **Atrioventricular septal defect (endocardial cushion defect)** | 745.60x, 745.61x, 745.69x | Q21.2x |
| **Coarctation of the aorta** | 747.10x | Q25.1x |
| **Common truncus (truncus arteriosus or TA)** | 745.0x | Q20.0x |
| **Double outlet right ventricle (DORV)** | 745.11x | Q20.1x |
| **Ebstein anomaly** | 746.2x | Q22.5x |
| **Hypoplastic left heart syndrome** | 746.7x | Q23.4x |
| **Interrupted aortic arch (IAA)** | 747.11x | Q25.2x, Q25.4x |
| **Pulmonary valve atresia and stenosis** | 746.01x (atresia),  746.02x (stenosis) | Q22.0x, Q22.1x |
| **Single Ventricle** | 745.3x | Q20.4x |
| **Tetralogy of Fallot (TOF)** | 745.2x | Q21.3x |
| **Total anomalous pulmonary venous connection (TAPVC)** | 747.41x | Q26.2x |
| **Transposition of the great arteries (TGA)** | 745.10x, 745.12x, 745.19x | Q20.3x, Q20.5x |
| **Tricuspid value atresia and stenosis** | 746.1x | Q22.4x |
| **Orofacial** |  |  |
| **Choanal atresia** | 748.0x | Q30.0x |
| **Cleft lip with cleft palate** | 749.2x | Q37.0x — Q37.9x |
| **Cleft lip alone (without cleft palate)** | 749.1x | Q36.0x — Q36.9x |
| **Cleft palate alone (without cleft lip)** | 749.0x | Q35.1x — Q35.9x |

Table 1. Continued

| **Gastrointestinal** |  |  |
| --- | --- | --- |
| **Biliary atresia** | 751.61x | Q44.2x — Q44.3x |
| **Esophageal atresia/tracheoesophageal fistula** | 750.3x | Q39.0x — Q39.4x |
| **Rectal and large intestinal atresia/stenosis** | 751.2x | Q42.0x — Q42.9x |
| **Small intestinal atresia/stenosis** | 751.1x | Q41.0x — Q41.9x |
| **Genitourinary** |  |  |
| **Bladder exstrophy** | 753.5x | Q64.10x, Q64.19x |
| **Cloacal exstrophy** | 751.5x | Q64.12x |
| **Congenital Posterior Urethral Valves** | 753.6x | Q64.2x |
| **Hypospadias** | 752.61x | Q54.0x—Q54.9x  Excluding Q54.4x |
| **Renal agenesis/hypoplasia** | 753.0x | Q60.0x — Q60.6x |
| **Musculoskeletal** |  |  |
| **Clubfoot** | 754.51x, 754.70x | Q66.0x, Q66.89x |
| **Craniosynostosis** | No specified code | Q75.0x |
| **Diaphragmatic hernia** | 756.6x | Q79.0x, Q79.1x |
| **Gastroschisis** | 756.73x, 756.79x | Q79.3x |
| **Limb deficiencies (reduction defects)** | 755.2x — 755.4x | Q71.0x — Q71.9x, Q72.0x — Q72.9x, Q73.0x — Q73.8x |
| **Omphalocele** | 756.72, 756.79x | Q79.2x |
| **Chromosomal** |  |  |
| **Deletion 22 q11** | 758.32x | Q93.81x |
| **Trisomy 13** | 758.1x | Q91.4x — Q91.7x |
| **Trisomy 18** | 758.2x | Q91.0x — Q91.3x |
| **Trisomy 21 (Down syndrome)** | 758.0x | Q90.0x — Q90.9x |
| **Turner Syndrome** | 758.6x | Q96.0x — Q96.9x |

NOTE: x means included all decimal places after this (e.g. for Encephalocele: code is 742.0x, so individuals with 742.0, 742.01, 742.02, 742.03, 742.04, etc. would be counted as having this major congenital anomaly)

Table 2. Imputed Versus Complete Datasets

|  | **Number imputed**  **n (%)** | **Population with complete data**  **(N = 2,568 – number imputed)**  **n (%)** | **Total population**  **N = 2,568**  **n (%)** | **P-value^†^** |
| --- | --- | --- | --- | --- |
| **Chorioamnionitis** | 2 (0.078%) | 158 (6.16%) | 158 (6.15%) | 0.9943 |
| **Hypertension during pregnancy** | 2 (0.078%) | 654 (25.49%) | 655 (25.51%) | 0.9875 |
| **Diabetes during pregnancy** | 2 (0.078%) | 512 (19.95%) | 513 (19.98%) | 0.9833 |
| **Maternal first trimester BMI^‡^** | 1558 (60.67%) | 28.74 (7.93) | 28.94 (7.91) | 0.4800 |
| **Birth length (cm)^‡^** | 651 (25.35%) | 42.78 (5.76) | 42.90 (5.78) | 0.4907 |
| **Head circumference (cm)^‡^** | 862 (33.57%) | 29.88 (3.78) | 29.96 (3.80) | 0.4707 |
| **1-minute Apgar score^‡^** | 27 (1.05%) | 6 (2) | 6 (2) | 0.9410 |
| **Length of birth hospitalization stay (days)^‡^** | 2 (0.078%) | 35.56 (38.17) | 35.55 (38.16) | 0.9912 |

Abbreviations: BMI, body mass index; cm, centimeters

^†^P-values were calculated using Chi-Square Tests, Fisher’s Exact Tests, and T-Tests

**^‡^**Data are expressed as mean ± standard deviation

Table 3. Sensitivity Analyses Based on Follow-Up Time

|  | **Overall model**  **N = 2,568** | | | **At least 6 months of follow-up**  **N = 2,084** | | | **At least 1 year of follow-up**  **N = 1,633** | | | **At least 2 years of follow-up**  **N = 612** | | |
| --- | --- | --- | --- | --- | --- | --- | --- | --- | --- | --- | --- | --- |
| **Variable** | **Parameter estimate(s)** | **SE** | **P-value** | **Parameter estimate(s)** | **SE** | **P-value** | **Parameter estimate(s)** | **SE** | **P-value** | **Parameter estimate(s)** | **SE** | **P-value** |
| **Intercept** | 11.02142 | 5.68115 | 0.0525 | 5.22533 | 7.14588 | 0.4647 | -2.24043 | 9.19100 | 0.8074 | 8.33585 | 15.82974 | 0.5987 |
| **Maternal race**  **Asian**  **Black/AA**  **Multiple races**  **Other**  **Unknown** | -0.15719  0.00283  -0.09668  -0.00151  -0.02251 | 0.03980  0.02518  0.05513  0.02850  0.04450 | <0.0001*  0.9105  0.0796  0.9579  0.6130 | -0.19380  0.00557  -0.11510  -0.00380  -0.02781 | 0.04880  0.03115  0.06861  0.03365  0.05428 | <0.0001*  0.8582  0.0936  0.9101  0.6085 | -0.22072  0.01588  -0.13550  -0.00386  -0.06738 | 0.05658  0.03746  0.08229  0.04090  0.06918 | <0.0001*  0.6717  0.0998  0.9248  0.3302 | -0.24621  -0.01897  -0.16138  -0.01130  -0.02404 | 0.08891  0.05345  0.12931  0.06007  0.10558 | 0.0058*  0.7228  0.2125  0.8509  0.8199 |
| **Maternal first trimester BMI**  **Underweight**  **Overweight**  **Obese** | -0.10882  0.01170  0.03154 | 0.05894  0.01860  0.01762 | 0.0650  0.5294  0.0736 | -0.13358  0.01428  0.03890 | 0.07351  0.02214  0.02111 | 0.0693  0.5190  0.0655 | -0.16403  0.02744  0.03615 | 0.09400  0.02644  0.02533 | 0.0812  0.2994  0.1536 | -0.17429  0.07928  0.08754 | 0.13059  0.04148  0.03857 | 0.1825  0.0565  0.0236* |
| **Maternal hypertension during pregnancy** | -0.00294 | 0.01909 | 0.8777 | -0.00742 | 0.02300 | 0.7472 | -0.00994 | 0.02799 | 0.7224 | -0.04096 | 0.04463 | 0.3591 |
| **Preeclampsia** | 0.00653 | 0.01859 | 0.7256 | 0.00819 | 0.02232 | 0.7137 | 0.01166 | 0.02702 | 0.6661 | 0.03933 | 0.04068 | 0.3341 |
| **Birth year** | -0.00552 | 0.00282 | 0.0507 | -0.00268 | 0.00355 | 0.4503 | 0.00093 | 0.00456 | 0.8385 | -0.00431 | 0.00786 | 0.5833 |
| **Multiple(s)** | 0.02730 | 0.01593 | 0.0867 | 0.03153 | 0.01908 | 0.0986 | 0.02857 | 0.02314 | 0.2172 | 0.03225 | 0.03464 | 0.3522 |
| **Gestational age (weeks)** | 0.01599 | 0.00569 | 0.0049* | 0.02260 | 0.00675 | 0.0008* | 0.03420 | 0.00817 | <0.0001* | 0.04858 | 0.01237 | <0.0001* |
| **Birth weight (gr)** | -0.00002 | 0.00003 | 0.4994 | -0.00003 | 0.00003 | 0.3234 | -0.00005 | 0.00004 | 0.1656 | -0.00004 | 0.00006 | 0.4707 |
| **Birth length (cm)** | -0.00022 | 0.00337 | 0.9469 | -0.00057 | 0.00411 | 0.8897 | -0.00011 | 0.00511 | 0.9821 | -0.00351 | 0.00804 | 0.6627 |
| **Head circumference (cm)** | -0.00915 | 0.00529 | 0.0837 | -0.01175 | 0.00652 | 0.0719 | -0.01654 | 0.00782 | 0.0345* | -0.02915 | 0.01191 | 0.0146* |
| **TPN** | -0.02370 | 0.02119 | 0.2634 | -0.03240 | 0.02569 | 0.2074 | -0.02544 | 0.03139 | 0.4178 | 0.01332 | 0.04892 | 0.7855 |
| **Received dextrose** | 0.03910 | 0.01818 | 0.0316* | 0.04628 | 0.02134 | 0.0302* | 0.05541 | 0.02494 | 0.0264* | 0.01119 | 0.03752 | 0.7657 |
| **Length of stay (days)** | -0.00029 | 0.00037 | 0.4378 | -0.00029 | 0.00042 | 0.4944 | -0.00022 | 0.00048 | 0.6510 | 0.000001 | 0.00065 | 0.9991 |

Abbreviations: AA, African American; BMI, body mass index; cm, centimeters; gr, grams; SE, standard error; TPN, total parenteral nutrition

Referent groups: White/Caucasian maternal race, normal maternal first trimester BMI, no hypertension, no preeclampsia, singleton, no TPN, no dextrose

*p<0.05. P-values were calculated using multivariable linear regression
